# Minimally invasive resection of synchronous thoracic esophageal and gastric carcinomas followed by reconstruction: a case report

**DOI:** 10.1186/s40792-015-0018-4

**Published:** 2015-02-04

**Authors:** Masayuki Honda, Hiroyuki Daiko, Takahiro Kinoshita, Takeo Fujita, Hidehito Shibasaki, Toshiro Nishida

**Affiliations:** Department of Gastrointestinal Oncology, Esophageal Surgery Division, National Cancer Center Hospital East, 6-5-1, Kashiwanoha, 277-0822 Kashiwa, Chiba Japan; Department of Gastrointestinal Oncology, Gastric Surgery Division, National Cancer Center Hospital East, 6-5-1, Kashiwanoha, 277-0882 Kashiwa, Chiba Japan

**Keywords:** TSEP, LTG, Minimally invasive surgery, Synchronous carcinomas, Laparoscopy-assisted colonic reconstruction

## Abstract

We report on a case of synchronous carcinomas of the esophagus and stomach. A 68-year-old man was referred to our hospital for an abnormality found during his medical examination. Further evaluation revealed squamous cell carcinoma in the thoracic lower esophagus and gastric adenocarcinoma located in the middle third of the stomach. Thoracoscopic esophagectomy in the prone position (TSEP), laparoscopic total gastrectomy (LTG) with three-field lymph node dissection, and laparoscopically assisted colon reconstruction (LACR) were performed. The patient did not have any major postoperative complications. His pathological examination revealed no metastases in 56 harvested lymph nodes and no residual tumor. He was followed up for 30 months without recurrence. To our knowledge, this is the first report of esophageal and gastric synchronous carcinomas that were successfully treated with a combination of TSEP, LTG, and LACR. These operations may be a feasible and appropriate treatment for this disease.

## Background

Minimally invasive surgical techniques for malignancy of the alimentary tract have been established as a treatment for esophageal, gastric, and colonic cancers [1-8]. First, in the treatment of esophageal carcinoma, thoracoscopic esophagectomy in the prone position (TSEP) is one of several minimally invasive esophageal surgeries performed to reduce significant surgical complications [9]. TSEP with extended lymphadenectomy is also a feasible and appropriate surgical technique for clinical stage I thoracic esophageal carcinoma [10]. Second, in the treatment of gastric carcinoma, laparoscopic total gastrectomy (LTG) with lymph node dissection is a minimally invasive surgery mainly for early stage gastric cancer in Japan. The incidence of operative complications is the same as that with conventional open surgery. LTG has advantages over open surgery, including earlier recovery, a shorter hospital stay, less pain, and better cosmetics [11]. Finally, in the treatment of colonic cancer, laparoscopic colectomy is widely used in cases of malignancy, which is supported by early data from several large randomized, controlled trials [12]. Thus, a laparoscopic surgical technique involving mobilization of the colon, which is useful in laparoscopically assisted colon reconstruction (LACR), has been established. However, to our knowledge, there is no report on the combination of minimally invasive surgical techniques such as TSEP, LTG, and LACR for treating synchronous carcinomas. Therefore, this is the first case report in which TSEP for early stage esophageal carcinoma, LTG with three-field lymph node dissection for gastric carcinoma, and LACR were performed simultaneously and successfully.

## Case presentation

**Figure 1:**
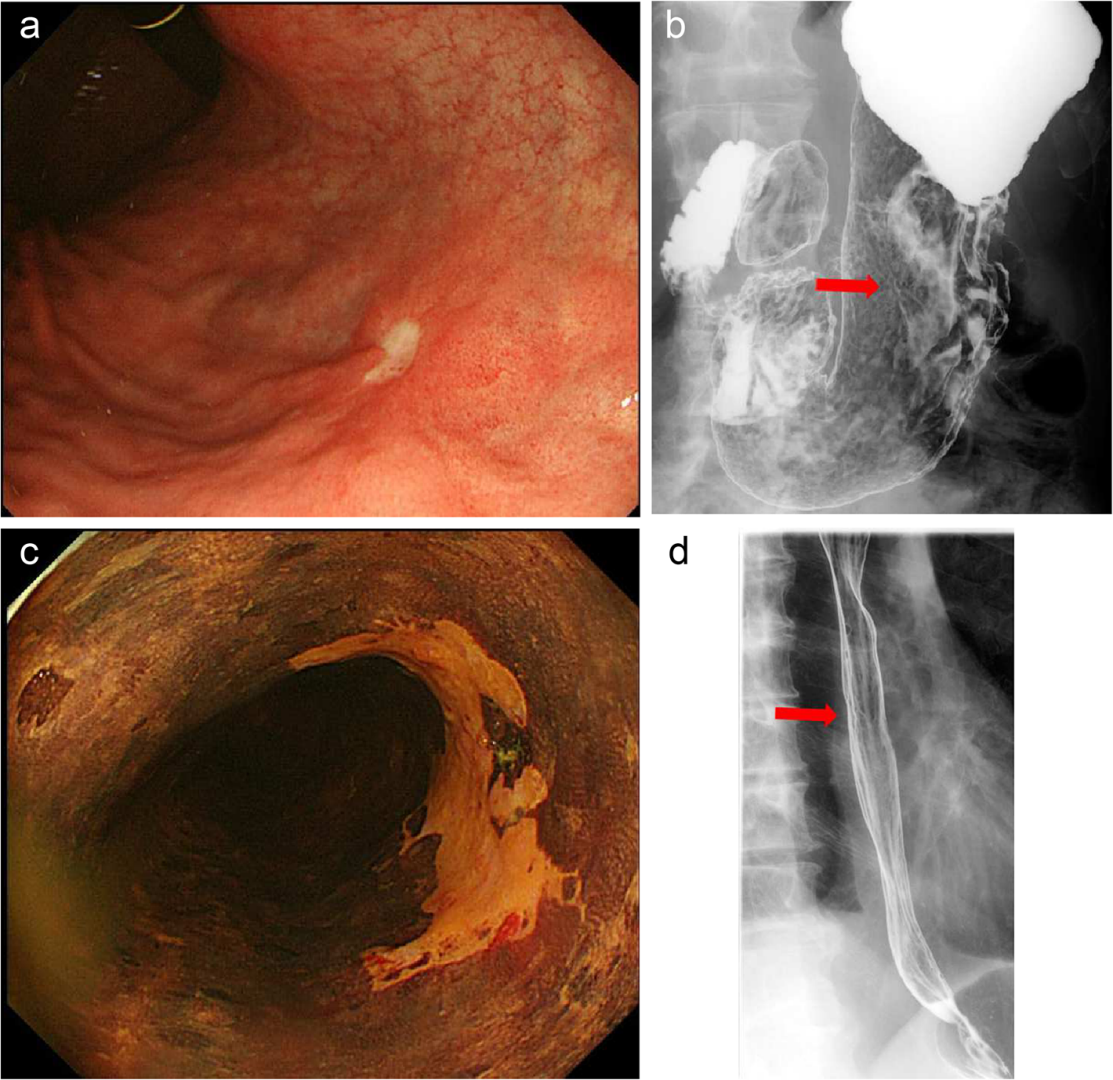
**Findings of the upper gastrointestinal series. (a,b)** Types 0-IIc and 0-III tumors are shown, with ulceration at the posterior wall of the middle third of the stomach. **(c,d)** A type 0-IIc tumor (right half-circumferential) is shown in the lower third of the esophagus.

A 68-year-old asymptomatic man was referred to our hospital for evaluation because of an irregularity in the gastric body, which was detected during a medical examination. His blood tests revealed no abnormalities, except for an elevated HbA1c level of 6.9%. Endoscopy and upper gastrointestinal series showed not only a type 0-IIc and 0-III gastric tumor (25 mm) with an ulceration at the posterior wall of the middle third of the stomach (Figure 1a and b) but also a type 0-IIc tumor (right, half-circumferential; 30 mm) in the lower third of the esophagus (Figure 1c,d). The biopsies of the tumors revealed squamous cell carcinoma in the thoracic lower esophagus (cT1bN0M0, cStageIA) and well- and moderately differentiated adenocarcinoma of the stomach (T1bN0M0, cStageIA). A computed tomography (CT) scan showed no primary tumor in the stomach or esophagus, no lymph node metastasis, and no tumors in other organs such as the liver and lungs. Colonoscopy and CT scan were performed to evaluate the colon and the patency of the middle colic vessels. Two polyps were detected in the ascending colon, and endoscopic polypectomy was performed 2 days prior to tumor resection.

The patient chooses TSEP for clinical stage I esophageal cancer among the therapies of TSEP, chemoradiotherapy or proton therapy, and informed consent was obtained.

### Tumor surgery

#### Thoracoscopic esophagectomy in the prone position

**Figure 2:**
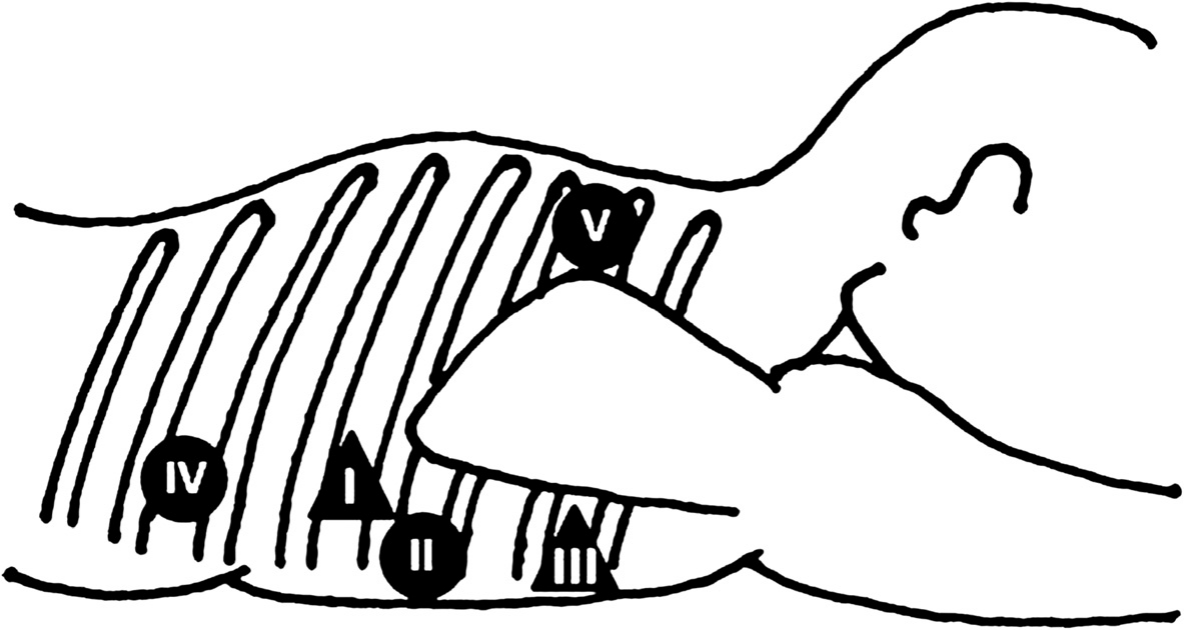
**Placement of the five ports for TSEP.** Filled circle, 5-mm port; filled triangle, 12-mm port).

TSEP was performed thoracoscopically, as previously described [10], with the patient intubated under epidural and general anesthesia. Five chest trocars were introduced (Figure 2), and carbon dioxide (CO_2_) was insufflated at a pressure of 8 mmHg to expand the mediastinum, maximizing the exposure of the intrathoracic esophagus without the need for additional retraction of the surrounding structures.

TSEP with mediastinal lymph node dissection involved three steps. First, a dissection of the middle to lower mediastinal lymph nodes was performed. The esophagus was circumferentially mobilized from the descending aorta, pericardium, and the left mediastinal pleura. The vagal trunk was cut below the level of its pulmonary branch, and the thoracic duct was preserved. Second, the procedure transitioned to the upper thorax. The arch of the azygos vein was cut using the linear stapler, and the right recurrent laryngeal nerve was identified just caudal to the right subclavian artery to ensure preservation. The fatty tissue containing lymph nodes around this area was dissected, and the right recurrent laryngeal nerve up to the inferior border of the thyroid gland was preserved. The esophagus was retracted by pulling the taped thread around the upper third of the esophagus, and *en bloc* dissection of the lymph nodes was performed by using scissors to prevent injury to the left recurrent laryngeal nerve below the aortic arch to the inferior border of the thyroid gland - no electrical or heat-producing devices were used. Finally, after the upper third of the esophagus was mobilized circumferentially, the esophagus was divided at the level of the arch of the azygos vein by linear stapling, and the esophagus was dissected by exposing the left side of the mediastinal pleura by retracting the anal stump. After complete mobilization of the esophagus, the subcarinal and bilateral bronchial lymph nodes were dissected completely. After the thoracoscopic procedures were completed, a chest tube was inserted.

#### Laparoscopic total gastrectomy

**Figure 3:**
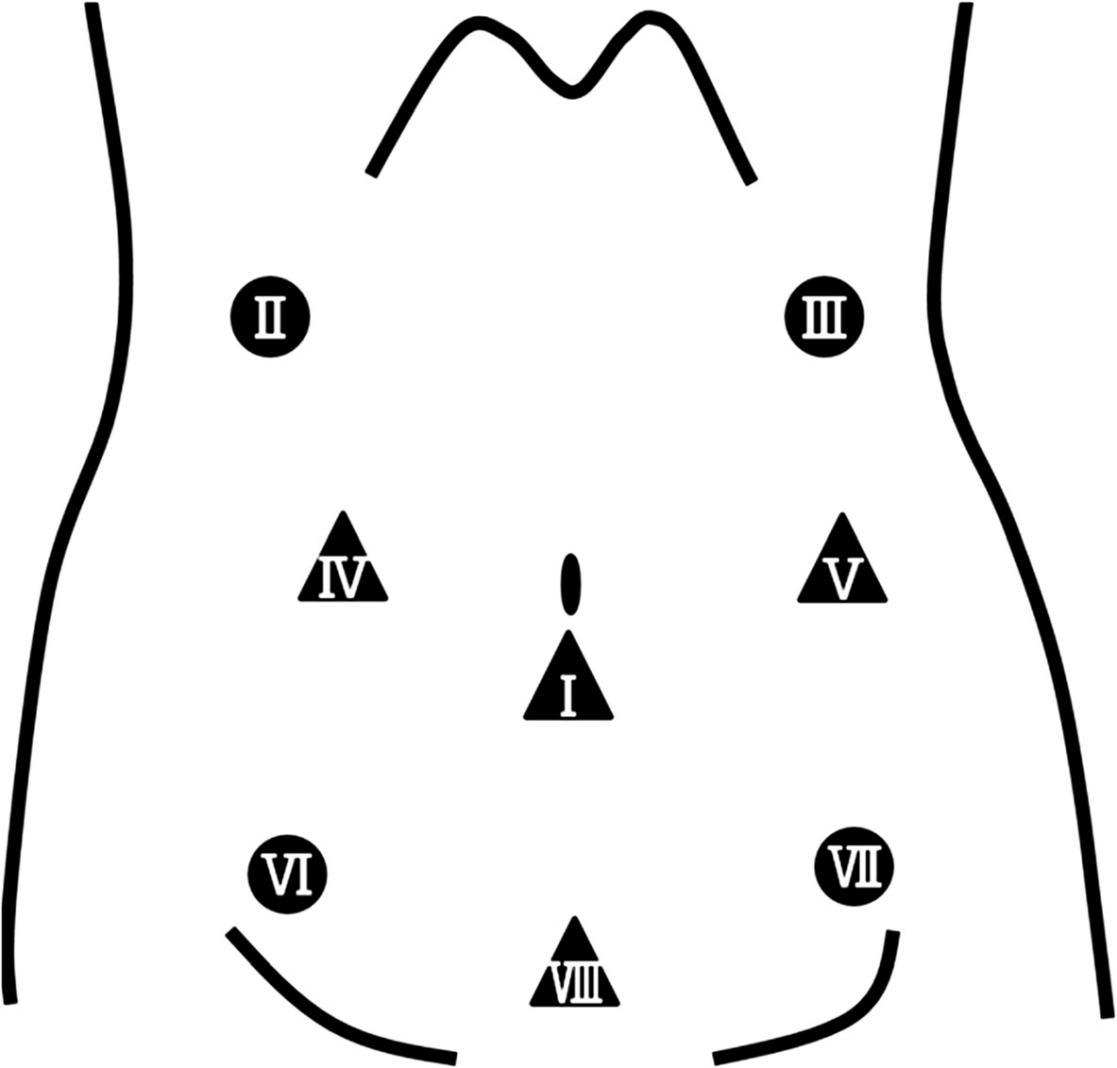
**Placement of the five ports (I ~ V) for LTG and the added ports (VI ~ VIII) for LACR.** Filled circle, 5-mm port; filled triangle, 12-mm port).

The position of patient was changed to the supine position under general anesthesia without a blocking balloon for double-lung ventilation. After five abdominal trocars were introduced (Figure 3) and CO_2_ was insufflated at a pressure of 10 mmHg to expand the abdomen, LTG with D1+ lymphadenectomy according to the Japanese Gastric Cancer treatment guidelines 2010 [13] was performed in five steps. First, the left and right greater omentum and lymph nodes were dissected along the gastroepiploic and infrapyloric vessels. Second, the duodenum, just distal to the pyloric ring, was transected by linear stapling. Third, the left lobe of the liver was retracted using a Penrose drain to expose the anatomy around the esophagogastric junction, as reported by Sakaguchi et al. [14]. Fourth, the suprapyloric nodes and nodes along the left gastric artery, common hepatic artery, splenic artery, and celiac artery were dissected. Finally, the abdominal esophagus was exposed after full mobilization of the stomach was achieved.

#### Laparoscopically assisted colon reconstruction

**Figure 4:**
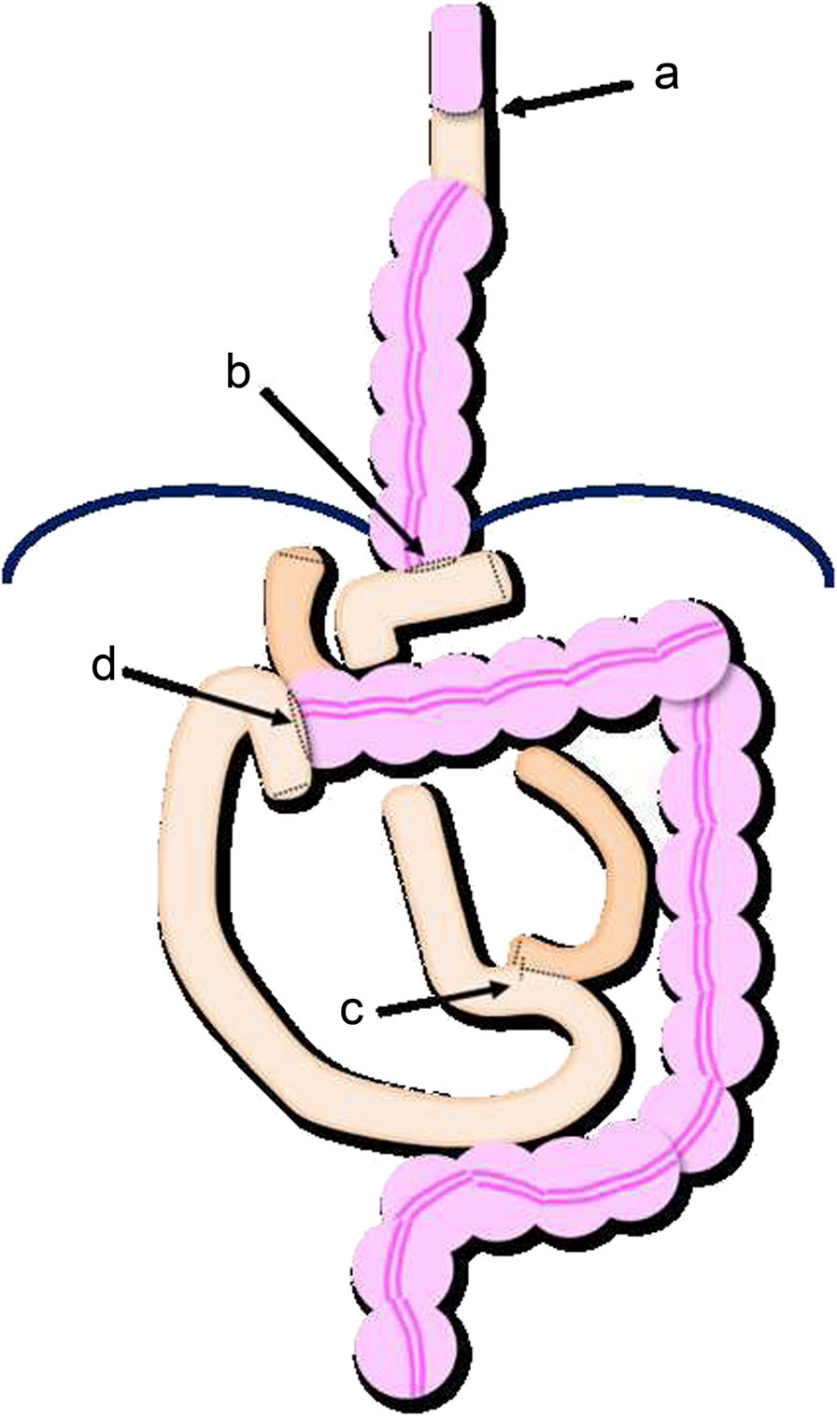
**Schematic diagram of TSEP, LTG, and LACR.** Ileocolic interposition was performed via the posterior mediastinum, and there are four anastomoses: **(a)** esophago-ileo hand-sewn anastomosis, **(b)** colo-jejunal anastomosis with a circular stapler, **(c)** jejuno-jejunal anastomosis with a linear stapler, and **(d)** ileocolic anastomosis with a circular stapler.

We added another three trocars, and the right colon was mobilized (Figure 3). After the infraumbilical incision was extended to 40 mm, the esophageal and gastric tumors were removed via mini-laparotomy simultaneously. The terminal ileum was divided into 4 cm proximally to the ileocecal valve by linear stapling, and the ileocolic vessels and accessory colic vein were divided. The blood supply for the right and transverse colon segments was from the middle colic vessels and there was innately no right colic artery and vein. Colonic interposition and Roux-en-Y colo-jejunal reconstruction were performed via the posterior mediastinum (Figure 4). During the abdominal approach, cervical lymph node dissection was performed in parallel.

Five chest trocar incisions, a 4-cm mini-laparotomy in the middle of the abdomen, and another seven abdominal trocar incisions were required. The operation was 545 min long, and blood loss was 95 g. Postoperatively, the patient did not have any major complications. However, there was a paralytic ileus, which was relieved using conservative treatment with an ileus tube. The pathological examination revealed no metastases in 56 harvested lymph nodes and no residual tumor. He was discharged 29 days postoperatively and was followed up for 30 months without any indications of recurrence or distant metastases.

## Discussion

For esophageal invasive carcinoma, open transthoracic esophagectomy is accepted as the best oncologic operation [15], partly because it allows the most extensive lymphadenectomy [16]. However, conventional transthoracic esophagectomy and transhiatal esophagectomy have high rates of morbidity (50%) and mortality (10%) [17]. Therefore, minimally invasive techniques, such as the combination of open surgery with either thoracoscopy or laparoscopy, are being used to reduce these complications [18,19].

A large systematic review reiterated that evidence is still lacking to confirm any real benefit in terms of long-term outcome, safety, and oncological quality with minimally invasive techniques [20,21]. However, our group reported a pilot study of the technical and oncological feasibility of TSEP for clinical stage I thoracic esophageal carcinoma. In the present case, TSEP with mediastinal lymph node dissection was performed with no residual tumor and no thoracic complications.

Regardless, in the treatment of early gastric carcinoma, the safety and efficacy of laparoscopic gastrectomy have been demonstrated in many clinical studies [22-24]. An increasing number of laparoscopic gastrectomies are currently being performed, especially in Eastern countries, which have high incidences of gastric carcinoma. For treating early gastric carcinoma with ulceration in the distal third of the stomach, distal gastrectomy with D1 or D1+ lymph node dissection is standard in Japan [13]. Yet, esophagectomy and distal gastrectomy were required for the double carcinomas in the present case, and total gastrectomy was performed. We used five ports in the upper or middle abdomen for LTG with D1+ lymph node dissection; however, the procedure for mobilizing the right colon was very difficult using just those ports, so additional three ports were added in the lower abdomen (Figure 3).

To our knowledge, there is only one case report on laparoscopic and thoracoscopic Ivor Lewis esophagectomy with colonic reconstruction for the treatment of a large esophagogastric carcinoma involving the gastric body and distal esophagus [25]. Therefore, this is the first report on synchronous carcinomas of the esophagus and stomach which were successfully treated using endoscopic surgeries (i.e., TSEP for esophageal carcinoma, LTG for gastric carcinoma, and LACR).

## Conclusions

In summary, we describe the first case of esophageal and gastric synchronous carcinomas treated using TSEP, LTG, and LACR. This operation can be used in patients with early stage cancers, because in our experience, it is feasible and appropriate.
